# Stability and Solution Structure of Binary and Ternary Cu(II) Complexes with l-Glutamic Acid and Diamines as Well as Adducts in Metal-Free Systems in Aqueous Solution

**DOI:** 10.1007/s10953-014-0269-x

**Published:** 2014-11-26

**Authors:** Romualda Bregier-Jarzebowska

**Affiliations:** Faculty of Chemistry, A. Mickiewicz University, Umultowska 89b, 61-614 Poznan, Poland

**Keywords:** Copper(II), l-Glutamic acid, Diamines, Molecular complexes, Inversion effect, Mixed complexes

## Abstract

Binary and ternary complexes of copper(II) with l-glutamic acid (Glu) and diamines 1,3-diaminopropane and 1,4-diaminobutane, putrescine (tn, Put), as well as adducts formed in the metal-free systems, have been investigated in aqueous solutions. The types of complexes formed and their overall stability constants were established on the basis of computer analysis of potentiometric results. The reaction centers and the modes of interaction were identified on the basis of spectroscopic studies (NMR, Vis and EPR). In the ligands studied the interaction centers are the oxygen atoms from carboxyl groups, nitrogen atom from the amine group of glutamic acid and the nitrogen atoms from amine groups of the diamines. The centers of noncovalent interaction in the adducts that formed in the metal-free systems are also potential sites of metal ion coordination, which is important in biological systems. In the Glu–diamine systems, molecular complexes of the (Glu)H_x_(diamine) type are formed. In the (Glu)H_2_(tn) adduct, in contrast to the corresponding complex with Put, an inversion effect was observed in which the first deprotonated amine group of tn became a negative reaction center and interacted with the protonated amine groups from Glu. Depending on the pH, the amine groups from the diamine can be either a positive or a negative center of interaction. In the Cu(Glu)_2_ species the first molecule of Glu takes part in metallation through all functional groups, whereas the second molecule makes a “glycine-like” coordination with the Cu(II) ions that is only through two functional groups. According to the results, introduction of Cu(II) ions into metal-free systems (Glu–diamine) changes the character of interactions between the bioligands in the complexes that form in Cu(II)–Glu–diamine systems and no ML…L′ type complexes are formed. However, in the ternary systems only the heteroligand complexes Cu(Glu)(diamine) and Cu(Glu)(diamine)(OH) are observed.

## Introduction

l-Glutamic acid is an amino acid present in many food products either in its free form or in peptides and proteins. This amino acid is found throughout the mammalian brain and participates in many metabolic pathways [1, 2]. Glutamic acid as well as glutamate receptors (GluRs) are excitatory neurotransmitters in the cerebral cortex (or central nervous system) [3–9]. As a neurotransmitter this compound is thought to play an important role in the functions of learning, memory and brain aging processes [10], but glutamic acid and related excitatory amino acids can also be toxic to central neurons. Excessive activation of GluRs during stress leads to the death of central neurons. The Glu neurotoxicity may also be involved in the genesis of various neurodegenerative diseases, physiology and pathology of brain functions [11–14]. l-Glutamic acids plays an important role in neuronal differentiation, migration and survival in the developing brain via facilitated Ca^2+^ transport [15] or in intestine metabolism [16]. Higher concentrations of glutamic acid have been noted in people suffering from Alzheimer’s [17–20], Parkinson’s diseases [21] and epilepsy [22].

Polyamines (PAs) [among them putrescine (Put), spermidine (Spd) and spermine (Spm)] are present in almost all living organisms. Protonated PAs are aliphatic cations with multiple functions and are essential for life. They are implicated in a variety of cellular processes, e.g. in embryonic development, chromatin organization, mRNA translation, ribosome biogenesis, cell growth and proliferation, programmed cell death, influence the transcriptional and translational stages of protein synthesis, stabilize membranes, modulate neurophysiological functions and may act as intracellular messengers. PAs stabilize nucleic acids and stimulate their replication [23–29]. They are found in high concentration in sperm fluid and in circulating blood (especially in erythrocytes) [30]. Polyamines accumulate in cancerous tissues and their concentration is elevated in body fluids of cancer patients [31]. PAs has implications in human diseases, for example parasite infection, gene therapy and diabetes [32]. Spermidine and spermine levels are usually less affected by stress while putrescine levels correlate closely with the density of cell necrosis [33]. Moreover, the mean level of spermidine is significantly increased over control values in the temporal cortex of patients with Alzheimer’s disease, while the putrescine level decreases [34].

Existing literature reports contain no information on studies investigating interactions occurring between glutamic acid and polyamines, or determining the influence of metal ions present in living organisms on interactions of this type. Hence, the study reported below, which is a follow-up of research conducted in the amino acid–PA and Cu(II)–amino acid–PA systems [35–37], presents the results of studies focused on Glu–diamine systems and relevant ternary systems with Cu(II) ions present in human cells [38, 39].

## Experimental

### Materials and Reagents

l-Glutamic acid (Glu), C_5_H_9_NO_4_ (purity 99 %), was purchased from Sigma–Aldrich and used without further purification. The compounds 1,3-diaminopropane (tn), C_3_H_10_N_2_ (purity 99 %), and 1,4-diaminobutane (Put), C_4_H_12_N_2_ (purity 99 %), were purchased from Sigma. The dinitrates of PAs were prepared by dissolving an amount of free amine and addition of an equimolar amount of HNO_3_. The white precipitate obtained was recrystallized, washed with methanol and dried in a desiccator over P_4_O_10_. The dinitrates of PAs were subjected to elemental analysis whose results (%C, %N, %H) are in agreement with the theoretically calculated values (±0.5 %). The elemental analysis was performed on an Elemental Analyzer CHN 2400, Perkin-Elmer. Copper(II) nitrate(V) trihydrate (purity p.a.) from POCH–Poland was purified by recrystallization from water. The complexometric method of determination of Cu(II) concentration was described earlier [40].

### Apparatus and Measuring Techniques

#### Equilibrium Study

Potentiometric studies were performed on a Methrom 702 SM Titrino with an autoburette. The glass electrode, Methrom 6.0233.100, was calibrated in terms of hydrogen ion concentration [41] with the preliminary use of borax (pH 9.225) and phthalate (pH 4.002) standard buffers. The concentrations of Glu and diamines were 1 × 10^−2^ mol·dm^−3^ in the metal-free systems and 2.6 × 10^−3^ mol·dm^−3^ in the systems with Cu(II). The ratio of ligand1:ligand2 (ligand1 = Glu, ligand2 = diamine) in the samples studied was 1:1, metal:ligand1 ranged from 1:1 to 1:2.6 in the binary systems, and metal:ligand1:ligand2 ranged from 1:1:1 to 1:2.6:2.6 in the ternary systems. Potentiometric titrations were performed at the constant ionic strength *μ* = 0.1 mol·dm^−3^ (KNO_3_) at *t* = 20 ± 1 °C under a neutral gas atmosphere (helium), using as titrant CO_2_-free NaOH solution (about 0.2 mol·dm^−3^). Addition of NaOH solution did not change the ionic strength, because the measurements were performed starting from the fully protonated polyamines, so the $- {\text{NH}}_{x}^{ + }$ cations were replaced by equivalent amounts of Na^+^. For each system a series of ten titrations was made; the initial volume of the sample was 30 cm^3^. No precipitate formation was observed in the entire pH range studied. The selection of the models and the determination of the stability constants of the complexes were made using either the SUPERQUAD [42] or HYPERQUAD [43] computer programs. The calculations were performed using 100–350 points for each experiment. Distribution of particular chemical forms was determined by the HALTAFALL system [44].

#### NMR Measurements

The samples for ^13^C NMR investigation were prepared by dissolving appropriate amounts of the ligands in D_2_O. DCl and NaOD were used to adjust the pD of solutions, correcting the pH readings (a pH meter N517 made by Mera-Tronik) according to the formula: pD = pH_readings_ + 0.40 [45]. The concentration of the ligands in the samples was 0.01 mol·dm^−3^, and the concentration ratios of Cu(II)-to-Glu and Cu(II)-to-Glu and diamines were 1:100 and 1:100:100 respectively. ^13^C NMR spectra were recorded on an NMR Gemini 300VT Varian spectrometer using dioxane as an internal standard. The positions of ^13^C NMR signals were converted to the TMS scale.

#### Vis Spectroscopy

The Vis spectra were taken on an Evolution 300 UV–Vis ThermoFisher Scientific spectrophotometer, for the same ligand concentrations as in the samples for potentiometric titrations, in a Plastibrand PMMA cell with 1 cm path length.

#### EPR Spectroscopy

The EPR spectra were recorded on an SE/X 2547 Radiopan spectrometer at 77 K in glass capillary tubes of 130 μL capacity. Solutions were prepared in a water:glycol mixture (3:1). The concentration of Cu(II) was 5 × 10^−3^ mol·dm^−3^ and that of the ligands was 1.3 × 10^−2^ mol·dm^−3^.

## Results and Discussion

Protonation constants of l-glutamic acid were determined and they are in good agreement with literature data (46–49). The sequence of deprotonation in Glu is –C_(1)_OOH, then –C_(5)_OOH and $- {\text{NH}}_{3}^{ + }$ groups [46, 49]. Copper(II) hydrolysis constants were taken from Ref. [50].

**Scheme 1:**
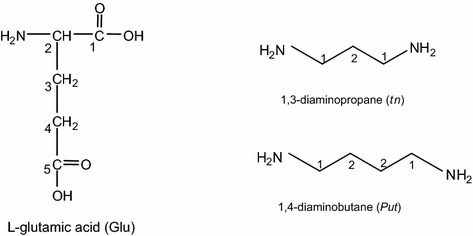
Chemical formulae of the bioligands studied

The structures of the ligands studied are presented in Scheme 1.

### Non-covalent Interactions in the Glu–tn and Glu–Put Systems

In the existing literature no information is available on noncovalent interactions in the metal-free systems of l-glutamic acid with diamines. In the systems studied the potential sites of weak interactions, leading to formation of molecular complexes, are the two carboxyl groups and the amine group in the Glu molecule and amine groups from tn and Put (Scheme 1). These centers are also potential sites of metal coordination by donor atoms, which is of importance in biological systems.

Dissociation of protons in the reaction:

$$ {\text{H}}_x({\text{Glu}}) + {\text{H}}_y({\text{PA}}) \rightleftharpoons ({\text{Glu}}) {\text{H}}^{(x+y-n)}({\text{PA}}) + n{\text{H}}^{+} $$

permits the use of computer analysis of potentiometric data for determination of the composition and stability constants of the adducts formed (as well as investigation of the coordination compounds) [51, 52]. The SUPERQUAD and HYPERQUAD programs use the nonlinear method of least squares to minimize the sum (*S*) of squares of residuals between the observed quantities (*f* ^obs^) and those calculated on the basis of the model $\left( {f^{\text{calc}} } \right){,}\,{\text{S}} = \sum\nolimits_{i = 1}^{n} {w_{i} } (f_{i}^{\text{obs}} - f_{1}^{\text{calc}} )^{2}$, where *n* is the number of measurements and *w*_*i*_ is the statistical weight.

Both in binary and ternary systems the testing usually begins with the simplest hypothesis and then in the next steps the models are expanded to include progressively more species, and the results are scrutinized to eliminate those species rejected by the refinement process. The criteria of the correct choice of a model are given in Ref. [51]. The modes of interactions were determined on the basis of the spectroscopic investigations in the pH range in which particular complexes dominate, as established on the basis of the equilibrium study.

#### Glutamic Acid–tn System

Ramaswamy and Murthy [53] have synthesized solid state complexes comprised of a single molecule of 1,3-propanodiamine and two molecules of l- or dl-glutamic acid and determined their structures. In the Glu–tn system studied in this work the following molecular complexes form in aqueous system as a result of noncovalent interactions: (Glu)H_4_(tn), (Glu)H_3_(tn) and (Glu)H_2_(tn), whose overall stability constants (log_10_ *β*) are 35.80, 32.38 and 22.58, respectively (Table 1).

**Table 1:** Statistical parameters (Σ, χ^2^), overall stability constants (log_10_ *β*) and equilibrium constants for adduct formation (log_10_ *K*_e_) in the Glu–tn and Glu–Put systems and GluH_*x*_ protonation constants

Systems	Species	Formation equilibria	Σ	χ^2^	log_10_ *β*	log_10_ *K*_e_
Glu^a^	H_3_Glu	H_2_Glu^2+^ + H^+^ ⇌ H_3_Glu^3+^	9.38	12.49	15.96 (1)	
	H_2_Glu	HGlu^+^ + H^+^ ⇌ H_2_Glu^2+^			13.69 (2)	
	HGlu	Glu + H^+^ ⇌ HGlu^+^			9.51 (1)	
Glu–tn	(Glu)H_4_(tn)	H_2_Glu + H_2_tn ⇌ (Glu)H_4_(tn)	11.87	14.31	35.80 (1)	2.47
	(Glu)H_3_(tn)	HGlu + H_2_tn ⇌ (Glu)H_3_(tn)			32.38 (1)	3.23
	(Glu)H_2_(tn)	HGlu + Htn ⇌ (Glu)H_2_(tn)			22.58 (1)	2.37
Glu–Put	(Glu)H_3_(Put)	HGlu + H_2_Put ⇌ (Glu)H_3_(Put)	16.59	19.89	31.62 (5)	1.60
	(Glu)H_2_(Put)	Glu + H_2_Put ⇌ (Glu)H_2_(Put)			22.80 (3)	2.29

Overall protonation constants of the diamines: Htn, log_10_ *β*_11_ = 10.70; H_2_tn, log_10_ *β*_12_ = 19.64 [54] and HPut, log_10_ *β*_11_ = 10.83; H_2_Put, log_10_ *β*_12_ = 20.51 [55]

^a^Literature values: HGlu (log_10_ *β*_11_), H_2_Glu (log_10_ *β*_12_), H_3_Glu (log_10_ *β*_13_): 9.59, 13.79, 15.97 [48]; 9.43, 13.50 [47]; 9.96, 14.28, 16.44 [50]; 9.51; 13.62; 15.74 [61]

As in the metal-free system with l-aspartic acid whose carbon chain is one methylene group shorter [35], the monoprotonated complex does not form (at least in a detectable amount), which supports the earlier suggestion that at least two centers of interactions in PA molecule are necessary for effective interaction and for ensuring formation of a stable adduct [56].

**Fig. 1:**
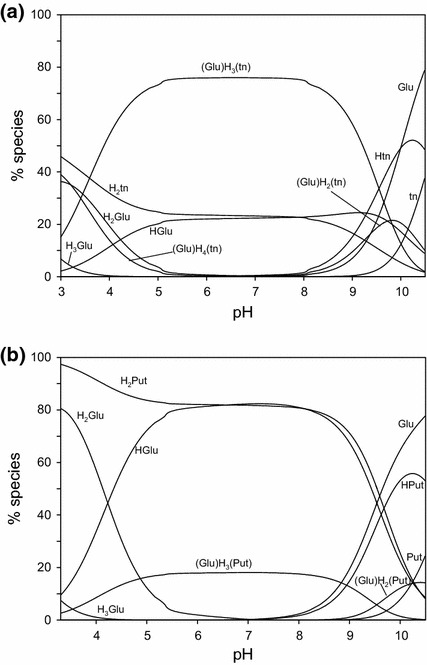
Distribution diagram for the Glu–tn and Glu–Put metal-free system where percentages of the species refer to the total amount of Glu: **a** Glu–tn: *C*_Glu_ = 1 × 10^−2^ mol·dm^−3^, *C*_tn_ = 1 × 10^−2^ mol·dm^−3^; **b** Glu–Put: *C*_Glu_ = 1 × 10^−2^ mol·dm^−3^, *C*_Put_ = 1 × 10^−2^ mol·dm^−3^

Figure 1a presents a distribution of adducts formed in the Glu–tn system. Below pH 4 the complex (Glu)H_4_(tn) is formed, and between pH 3.0 and 10.5 the (Glu)H_3_(tn) species is formed which reaches its highest concentrations in the pH range from 5 to 8. Above pH 8 formation of the adduct (Glu)H_2_(tn) was detected.

**Fig. 2:**
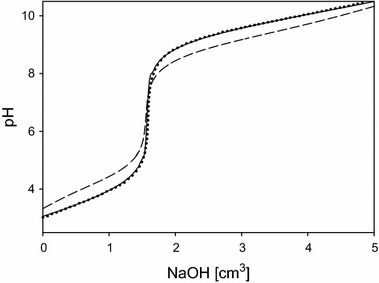
Experimental and simulated titration curves for the Glu–tn system (*C*_Glu_ = 1 × 10^−2^ mol·dm^−3^, *C*_tn_ = 1 × 10^−2^ mol·dm^−3^): *dotted line*—experimental curve; *solid line*—simulated curve (adduct formation was taken into account); *dashed line—*simulated curve (adduct formation was not taken into account

According to the earlier findings [56–58], the correct choice of the model is confirmed by overlapping of the experimental titration curves obtained from the equilibrium study with those obtained from computer simulations performed by taking into account formation of adducts (with use of the determined *β* values), Fig. 2. If the formation of adducts is disregarded, then the experimental and simulated curves are divergent.

As follows from the analysis of protonation constants of Glu and tn and the pH range of adduct formation (Fig. 1a), interactions in the (Glu)H_4_(tn) complex may involve the fully protonated amine groups as positive centers and a deprotonated –C_(1)_OO^−^ from the amino acid as a negative center. This mode of interaction follows from analysis of the ^13^C NMR spectra, Table 2.

**Table 2:** ^13^C NMR signal positions for the Glu–diamine systems and their changes in relation to the single ligands (ppm, in parentheses)

Systems	pH	Glu					PA	
		C_(1)_	C_(2)_	C_(3)_	C_(4)_	C_(5)_	C_(1)_	C_(2)_
Glu–tn	3.0	174.806 (0.201)	54.849 (0.057)	31.342 (0.040)	26.562 (0.037)	178.472 (0.033)	25.641 (0.077)	37.417 (0.012)
	6.0	175.354 (0.105)	55.456 (0.044)	34.259 (0.064)	27.744 (0.017)	182.083 (0.139)	25.654 (0.107)	37.424 (0.011)
	10.0	181.850 (0.720)	56.438 (0.090)	34.807 (0.033)	31.489 (0.024)	183.318 (0.125)	30.888 (0.090)	38.492 (0.040)
Glu–Put	6.0	175.347 (0.012)	55.476 (0.024)	34.273 (0.030)	27.737 (0.024)	182.347 (0.059)	39.687 (0.073)	24.679 (0.031)
	10.0	182.691 (1.561)	56.565 (0.217)	34.874 (0.100)	31.970 (0.105)	183.479 (0.905)	39.837 (2.129)	25.261 (0.645)

The shifts of signals assigned to carbon atoms of adducts in the ^13^C NMR spectra are related to the change in electron density on the atoms from the neighborhood of the centers involved in weak noncovalent interactions. No significant changes in the positions of signals assigned to the atoms farther from the interaction centers are noted. At pH 3, at which the dominant species is the adduct (Glu)H_4_(tn), the shift of the signal assigned to C(1) from 1,3-diaminopropane is 0.077 ppm, while the shift of the signal attributed to carbon atoms from deprotonated carboxyl group –C_(1)_OO^−^ of the amino acid is 0.201 ppm. Much smaller shifts occur for the signals assigned to C_(2)_ and C_(5)_ from Glu, 0.057 and 0.033 ppm, respectively (Table 2), mean that at this pH both the $- {\text{NH}}_{3}^{ + }$ and –C_(5)_OOH functional groups of Glu do not play a significant part in the interactions, which is well understood as these groups are protonated and blocked for the interaction with the positive centers in the polyamine molecule. Hence, a deprotonated carboxyl group from the amino acid and nitrogen atoms from the amino groups of diamine take part in the interactions in adduct (Glu)H_4_(tn). With increasing pH the second carboxyl group from the amino acid is deprotonated (a negative reaction center) and can take part in the interactions between bioligands in forming the dominant adduct (Glu)H_3_(tn).

The equilibrium constant of (Glu)H_3_(tn) of log_10_ *K*_*e*_ = 3.23 is higher than that of (Glu)H_4_(tn), log_10_ *K*_*e*_ = 2.47, which is a clear indication of participation of another active center in the former adduct and development of a favorable interaction between the bioligands in (Glu)H_3_(tn). Because of the different compositions of particular species, the overall stability constants (log_10_ *β*) cannot be directly applied in analyzing the character of these interactions. Therefore, the efficiency of bonding was estimated on the basis of the equilibrium constant calculations. In the ^13^C NMR spectrum at pH 6, at which the dominant species is (Glu)H_3_(tn), the signals assigned to –C_(5)_OO^−^ and –C_(1)_OO^−^ are shifted by 0.139 and 0.105 ppm, respectively, relative to their positions in the spectrum of the free ligand, which confirms the participation of both carboxyl groups of Glu in the interactions. The $- {\text{NH}}_{3}^{ + }$ group from the amino acid is excluded from the interactions in (Glu)H_3_(tn) as the shift of the signal assigned to C_(2)_ is only 0.044 ppm. The shift of the signal assigned to C(1) from the vicinity of the amine groups of tn, of 0.107 ppm, confirm the participation of these amine groups in the interactions between bioligands. The magnitude of the changes is of the same order as those observed earlier for similar systems with polyamines [56, 59].

**Fig. 3:**
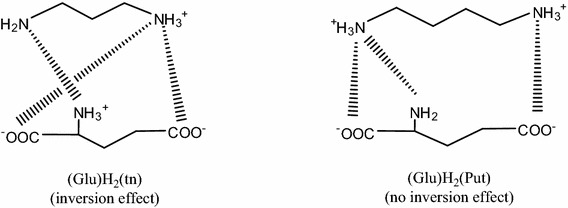
Tentative modes of interaction in the (Glu)H_2_(tn) and (Glu)H_2_(Put) adducts

The equilibrium constant of (Glu)H_2_(tn) adduct formation in the reaction HGlu + Htn ⇌ (Glu)H_2_(tn), log_10_ *K*_*e*_ = log_10_ *β*_(Glu)H2(tn) _− log_10_ *β*_HGlu_ − log_10_ *β*_Htn_ = 2.37, is smaller than that obtained for (Glu)H_3_(tn), log_10_ *K*_*e*_ = 3.23. The above changes in the equilibrium constants correlate well with the change in the mode of interaction in (Glu)H_2_(tn) which dominates at pH close to 10. When the first proton is dissociated from tn, a negative active center appears in the diamine molecule and it is involved in the interaction with a still (log_10_ *K*_1_ = 9.51) protonated amine group of Glu and the inversion effect takes place, similar as to (Asp)H_2_(tn) [35]. Thus, as a result of pH change, in the system studied one of the amine groups (deprotonated one) from tn becomes a negative reaction center and reacts with the protonated amine group from the amino acid. The amine group of the polyamine can be either a positive or a negative reaction center. The inversion effect is clearly indicated by analysis of changes in the ^13^C NMR spectrum, Table 2. At pH 10.0, the shift of signals assigned to C(1) from diamine is 0.090 ppm, while the shifts of the signals assigned to C_(1)_, C_(2)_ and C_(5)_ from Glu are 0.720, 0.090 and 0.125 ppm, respectively, and prove that the amine groups –NH_2_ and $- {\text{NH}}_{3}^{ + }$ from tn are involved in the interactions with $- {\text{NH}}_{3}^{ + }$ and two carboxyl groups from Glu, as suggested in Fig. 3.

As obtained from the molecular electronic structure calculation (program GAUSSIAN 03 [60], Ground State method/DFT/B3LYP/LAND2DZ level, solvation model), the partial charges on –NH_2_ and $- {\text{NH}}_{3}^{ + }$ in Htn are −0.007 and +0.601, respectively (in lower pH where H_2_tn exists the partial charges on both $- {\text{NH}}_{3}^{ + }$ groups are positive: +0.661), while for those on –C_(1)_OO^−^, –C_(5)_OO^−^ and $- {\text{NH}}_{3}^{ + }$ in HGlu the partial charges are −0.784, −0.830 and +0.523, respectively. These values are in a good agreement with the model following from the NMR investigation. Taking into account that both amino groups from tn and both carboxyl groups together with the amine group from Glu are involved in the interactions, the intramolecular interactions of the partial charges in the (Glu)H_2_(tn) adduct can be consistent only with the inversion model, Fig. 3. The inversion effect was first described in our earlier paper [35].

#### Glutamic Acid–Putrescine System

In the system Glu–Put, similar to Asp–Put [35], formation of only two adducts (Glu)H_3_(Put) and (Glu)H_2_(Put) is observed. The species (Glu)H_3_(Put) forms in the pH range from about 3 to 10, while the second adduct forms above pH 9 (Fig. 1b). Similarly, as with the corresponding system with aspartic acid [35], the concentrations of adducts with Put are much lower than those of the corresponding complexes with tn, which is in good agreement with the differences in their log_10_ *K*_*e*_ values (Table 1). This confirms our earlier conclusions [35–37] that the length of PA chain is an important factor influencing the stability of complexes. In the pH range of the (Glu)H_3_(Put) complex, the amine group from Glu is protonated and thus excluded from the interaction with the protonated $- {\text{NH}}_{3}^{ + }$ groups from Put, which is confirmed by analysis of the ^13^C NMR spectra. The signal assigned to C_(2)_ from the vicinity of the amine group is shifted only by 0.024 ppm.

The NMR signals assigned to C_(1)_ and C_(5)_ from l-glutamic acid (Table 2) at pH 6 are shifted by 0.012 and 0.059 ppm, which (based on the experience gathered in our previous studies [35–37, 57–59] ) means that only the –C_(5)_COO^−^ carboxyl group of this acid is involved in the interaction with putrescine. The signals assigned to C(1) from Put is significantly shifted by 0.073 ppm, which indicates that both protonated amine groups, as positive centers, are involved in the interaction with the amino acid.

In the range of high pH when another center is deprotonated, the (Glu)H_2_(Put) adduct is formed. Deprotonation of the amine group from Glu and dissociation of the first proton from Put take place practically in the same pH range (as follows from the protonation constants of the two ligands) (Table 1). The involvement of an additional active center in the interaction in the diprotonated adduct is evidenced by the higher equilibrium constant for formation of this species, log_10_ *K*_*e*_ = 2.46, than that for (Glu)H_3_(Put) formation, log_10_ *K*_*e*_ = 1.60. Although the differences in log_10_ *K*_*e*_ values between both adducts only correspond to small energy changes (about 4.8 kJ·mol^−1^), they are sufficient to demonstrate different modes of interaction in these forms, which is confirmed by ^13^C NMR spectra (Table 2).

At pH 10, where (Glu)H_2_(Put) is dominant, the signals assigned to C_(1)_, C_(5)_ and C_(2)_ from the amino acid molecule are shifted by 1.561, 0.905 and 0.217 ppm, respectively, while that assigned to C(1) from putrescine is shifted by 2.129 ppm. These shifts point to the involvement of all active centers from Glu and Put in the weak noncovalent interactions between the ligands in (Glu)H_2_(Put). Analysis of the IR spectra shows that the band at 3,398 cm^−1^, assigned to the stretching and deformation vibrations of $- {\text{NH}}_{3}^{ + }$ from Put recorded at pH 10, which is in the pH range in which (Glu)H_2_(Put) dominates, is not shifted relative to the corresponding band (3,399 cm^−1^) in the spectrum of (Glu)H_3_(Put). This observation means that in the molecular complex (Glu)H_2_(Put) both $- {\text{NH}}_{3}^{ + }$ groups from Put have not been deprotonated and thus cannot interact with the still protonated amine group of the amino acid, hence no inversion effect takes place.

The second protonation constant of Put is almost one log_10_ *K* unit higher than that for tn. The deprotonation of putrescine takes place above the pH range of deprotonation of glutamic acid, in contrast to the system of Glu–tn, which explains the differences in the mode of interaction in the adducts (Glu)H_2_(Put) and (Glu)H_2_(tn).

### Cu(II)–Glu Binary Systems

For the reliable assessment of results obtained in the ternary systems, measurements were also performed in the binary Cu(II)–Glu system under the same conditions, and the log_10_ *β* values determined for the complexes of Cu(II) ions with Glu are in good agreement with literature data [61–63]. Table 3 presents the overall stability constants (log_10_ *β*) and the equilibrium constants for formation (log_10_ *K*_*e*_) of the species in the studied binary system.

**Table 3:** Statistical parameters (Σ, χ^2^), overall stability constants (log_10_ *β*) and equilibrium constants (log_10_ *K*_e_) of complexes formed in the Cu(II)–Glu, Cu(II)–Glu–tn and Cu(II)–Glu–Put systems

Systems	Species	Formation equilibria	Σ	χ^2^	log_10_ *β*	log_10_ *K*_e_
Cu(II)–Glu	CuH(Glu)	Cu^2+^ + H^+^+ Glu ⇌ CuH(Glu)^3+^	17.12	18.75	13.03 (5)	3.51
	Cu(Glu)	Cu^2+^ + Glu ⇌ Cu(Glu)^2+^			8.52 (5)	8.52
	Cu(Glu)_2_	Cu^2+^ + 2Glu ⇌ Cu(Glu)_2_^2+^			15.01 (7)	6.49
	Cu(Glu)(OH)	Cu^2+^ + Glu + H_2_O ⇌ Cu(Glu)(OH)^2+^+ H^+^			1.85 (6)	–
Cu(II)–Glu–tn	Cu(Glu)(tn)	Cu^2+^+ Glu + tn ⇌ Cu(Glu)(tn)^2+^	13.19	34.56	18.26 (4)	9.74
	Cu(Glu)(tn)(OH)	Cu^2+^+ Glu +tn + H_2_O ⇌ Cu(Glu)(tn)(OH)^2+^+H^+^			9.60 (6)	–
Cu(II)–Glu– Put	Cu(Glu)(Put)	Cu^2+^+ Glu + Put ⇌ Cu(Glu)(Put)^2+^	9.26	14.91	16.56 (4)	8.04
	Cu(Glu)(Put)(OH)	Cu^2+^+Glu + Put + H_2_O⇌Cu(Glu)(Put)(OH)^2+^+H^+^			6.45 (5)	–

Overall stability constants (log_10_ *β*) of complexes in the binary systems: Cu(II)–tn: CuH(tn), 15.78; Cu(tn), 9.68; Cu(tn)_2_, 16.79; Cu(tn)_3_, 21.66 [54]; Cu(II)–Put: CuH(Put), 15.83; Cu(Put), 8.62; Cu(Put)_2_, 13.40; Cu(Put)_2_(OH), 0.065 [55]

The protonated species CuH(Glu) forms in the pH range from 2.5 to 6, while at pH 5.5 the dominant complex is Cu(Glu), binding about 80 % of the Cu(II) ions. The Cu(Glu)_2_ species occurs in the pH range from 5.5 to above 10 and it dominates in the pH range 7–9 where it binds more than 60 % of the Cu(II) ions. The hydroxo complex Cu(Glu)(OH) begins to form from pH 5. The UV–Vis and EPR parameters (Table 4) at pH 4, at which CuH(Glu) is dominant, are: *λ*_max_ = 765 nm, *g*_||_ = 2.280 and *A*_||_ = 170, respectively. As implied by these parameters, the oxygen atoms from deprotonated carboxyl groups of Glu take part in the coordination of Cu(II) ions, while the $- {\text{NH}}_{3}^{ + }$ group, which is protonated at this pH value, is blocked and does not take part in the interaction. These conclusions were drawn on the basis of analysis of spectroscopic data obtained for analogous systems [35–39].

**Table 4:** Visible and EPR spectral data for the Cu(II)–Glu–tn and Cu(II)–Glu–Put systems

Species	pH	*λ*_max_ (nm)	*ε* (L·mol^−1^·cm^−1^)	EPR	
				*g*_||_	*A*_||_ (10^−4^ cm^−1^)
CuH(Glu)	4.0	765	30	2.280	170
Cu(Glu)	5.5	714	35	2.279	190
Cu(Glu)_2_	8.0	616	62	2.221	205
Cu(Glu)(tn)	8.0	596	29	2.246	177
Cu(Glu)(tn)(OH)	10.5	593	32	2.282	179
Cu(Glu)(Put)	9.7	613	24	2.249	176
Cu(Glu)(Put)(OH)	10.5	610	25	2.289	189

The involvement of carboxyl groups in coordination of Cu(II) ions is confirmed by the shifts of the ^13^C NMR signals assigned to C_(1)_ and C_(5)_ from the carboxyl groups of the ligand, by 0.153 and 0.463 ppm, respectively (chromophore {2O} [64–67]). Taking into account the limitations in the use of NMR for investigation of paramagnetic ions, the NMR spectra of the species were recorded by the decoupling technique at low concentrations of metal ions. The pH ranges of the dominant complexes in the distribution diagram of the species are practically the same as for systems at higher concentrations of the metal ions and the ligands. Significant changes in the chemical shifts were observed only in the pH ranges in which the complexes were present (determined on the basis of potentiometric measurements). The results were also carefully verified with the equilibrium data and electronic spectra. A similar problem has been discussed earlier for other systems with Cu(II) ions [59, 67]. The increase in the equilibrium constant for formation of Cu(Glu) by 5.01 log_10_ *K*_*e*_ units relative to the value obtained for CuH(Glu) (Table 3) clearly points to the involvement of the already deprotonated amine group –NH_2_ from the amino acid in the inner coordination sphere of Cu(II) in Cu(Glu), which is consistent with the model proposed by Nagypal [61] and Sajadi [62]. Moreover, the d–d band in the visible spectrum *λ*_max_ is shifted towards higher energies, from 765 to 714 nm, while the EPR parameters take the values *g*_||_ = 2.279 and *A*_||_ = 190 (Table 4). All this confirms the above described mode of coordination in the Cu(Glu) complex, that is, the presence of the chromophore {N,2O}; the same mode of coordination has been reported for the Cu(Asp) complex [68]. The equilibrium constant of formation of the Cu(Asp) species, log_10_ *β* = log_10_ *K*_*e*_ = 8.76 [68], is slightly higher than that obtained for Cu(Glu), log_10_ *β* = log_10_ *K*_*e*_ = 8.52.

In the Cu(Glu) complex a seven-membered ring is formed, while in Cu(Asp) a six-membered one is present. The UV–Vis and EPR parameters for Cu(Glu)_2_, dominant at pH 8, are *λ*_max_ = 616 nm, *g*_||_ = 2.221 and *A*_||_ = 205 (Table 4), which point to the formation of a {2N,3O} chromophore. The first molecule of Glu takes part in metallation through all available reaction centers while the second one interacts with copper(II) in a “glycine-like” mode through two functional groups, i.e. the –NH_2_ amine group and oxygen atom from one carboxyl group. Moreover, in the ^13^C NMR spectrum at pH 8, the signals assigned to C_(1)_, C_(2)_ and C_(5)_ are shifted by 0.061, 0.180 and 0.132 nm, respectively. Thus, it can be assumed that in the Cu(Glu)_2_ binary complex, the donor atoms occupy five coordination sites of the Cu(II) ion, which is consistent with the model proposed by Nagypal [61], but inconsistent with the model developed by Li [69] in which nitrogen atoms from both Glu molecules and the oxygen atoms from only one –C_(1)_OO^−^ carboxyl group from each ligand are proposed to be involved in “glycine-like” coordination (chromophore {2N,2O}). The Cu(Glu)(OH) complex (similar to Cu(Asp)(OH) [69]) occurs in the same pH range as that of the domination of Cu(Glu)_2_ and, therefore, no spectra could be measured.

### Ternary Cu(II)–Glutamic Acid–Diamine Systems

The overall stability constants of mixed-ligand complexes formed in the ternary systems were calculated on the basis of the protonation constants of the ligands (Table 1), and overall stability constants (log_10_ *β*) of the binary species formed in the systems Cu(II)–tn [54], Cu(II)–Put [55] and Cu(Glu) (Table 3) determined earlier in the same conditions. The hydrolysis constants of Cu(II) ions were taken from [46].

#### Cu(II)–Glu–tn System

**Fig. 4:**
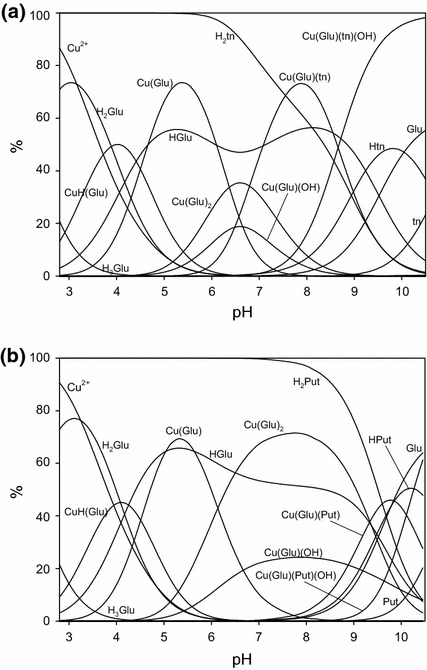
Distribution diagram for the Cu(II)–Glu–tn (**a**) and Cu(II)–Glu–Put (**b**) systems where percentages of the species refer to total amount of metal (*C*_Cu(II)_ = 1 × 10^−3^ mol·dm^−3^, *C*_Glu_ = 2.6 × 10^−3^ mol·dm^−3^, *C*_tn_ = 2.6 × 10^−3^ mol·dm^−3^, *C*_Put_ = 2.6 × 10^−3^ mol·dm^−3^)

In the ternary system Cu(II)–Glu–tn, the formation of Cu(Glu)(tn) and Cu(Glu)(tn)(OH) complexes was established on the basis of computer analysis of the potentiometric titration data (HYPERQUAD program). Table 3 presents the overall stability constants (log_10_ *β*) and the equilibrium constants of formation (log_10_ *K*_*e*_) of these complexes. The Cu(Glu)(tn) species is formed in the pH range from 6 to 10 and at pH 8 it binds about 80 % of the Cu(II) ions, Fig. 4a.

**Fig. 5:**
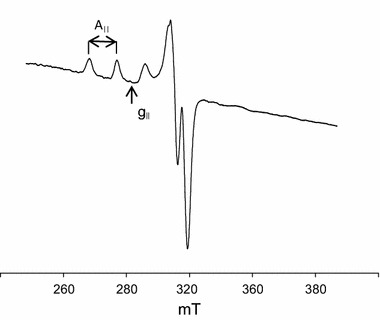
EPR spectra of the Cu(Glu)(tn) complex at pH 8

The energy of d–d transitions (*λ*_max_ = 596 nm) and EPR parameters (*g*_||_ = 2.246 and *A*_||_ = 177), Table 4 and Fig. 5, at pH 8 point to coordination through three nitrogen atoms and the oxygen atoms from Glu in the Cu(Glu)(tn) complex.

**Fig. 6:**
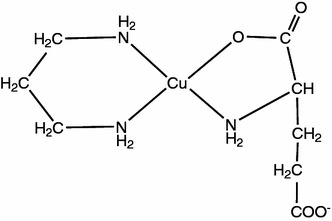
Tentative mode of coordination in the Cu(Glu)(tn) complex

There is a clear relation between the energy of d–d transition as well as EPR parameters and the number of donor atoms in the inner coordination sphere of Cu(II). For tetragonal and square pyramidal species, the ground state is normally d_x2−y2_ or rarely d_xy_. As earlier established for Cu–N_*x*_ (*x* = 1–6) and Cu–N_*x*_O_*y*_ (*x* = 0–4, *y* = 0–6), in planar bonding the value of *g*_||_ decreases and that of *A*_||_ increases [65, 70]. The involvement of three nitrogen atoms in metallation is also indicated by the ^13^C NMR spectra. The signal assigned to the C_(2)_ atom neighboring the –NH_2_ from l-glutamic acid is shifted by 0.070 ppm with respect to its position in the spectrum of the free ligand, while the signals assigned to C(1) and C(2) from tn are shifted by 1.070 and 0.500 ppm. Moreover, at the same pH, the signal corresponding to C_(1)_ from Glu is shifted by 0.069 ppm, while that corresponding C_(5)_ from Glu is shifted by 0.042 ppm. Therefore, in Cu(Glu)(tn), two nitrogen atoms from the diamine participate in coordination, while the coordination of glutamic acid is glycine-like only through two functional groups: nitrogen atom from the amino group and the –C_(1)_OO^−^ carboxyl group, as shown in Fig. 6.

The equilibrium constant of Cu(Glu)(tn) formation, log_10_ *K*_*e*_ = log_10_ *β*_Cu(Glu)(tn) − _log_10_ *β*_Cu(Glu)_ = 9.74 (Table 3), is similar to log_10_ *K*_*e*_ = 9.68 obtained for the binary Cu(tn) complex [54] (chromophore {2N}) and confirms the involvement of the two nitrogen atoms from diamine in the coordination with Cu(II) ions in Cu(Glu)(tn) (*K*_*e*_ corresponds to the energy of tn bonding with the metal). The hydroxo complex Cu(Glu)(tn)(OH) starts forming at pH close to 7.5 and at pH 10.5 it binds about 95 % of the Cu(II) ions (Fig. 4a). Its ^13^C NMR spectrum at pH 10.5 reveals that the signals assigned to C(1) from the diamine are shifted by 0.109 ppm, which indicates that nitrogen atoms from the amino groups of tn are involved in metallation. The quenching of signals assigned to C_(1)_ and C_(2)_ atoms from Glu, and a shift of the signal assigned to C_(5)_ from Glu, mean that in Cu(Glu)(tn)(OH) the coordination is realized through all donor centers of Glu, which was confirmed by the Vis and EPR results (Table 4).

#### Cu(II)–Aspartic Acid–Putrescine System

Similarly as in the system with tn, in the system with Put only two complexes are formed Cu(Glu)(Put) and Cu(Glu)(Put)(OH). The equilibrium constant for MLL’ species formation is log_10_ *K*_*e*_ = 8.04 (Table 3) and is similar to log_10_ *K*_e_ = 8.62 for formation of the binary complex Cu(Put) (chromophore {2N}) [55], which indicates involvement of both nitrogen donor atoms from Put in the metallation in Cu(Glu)(Put). This mode of coordination through two nitrogen atoms from PA amine groups is also suggested by the shifts of the C(1) Put signals in the ^13^C NMR spectrum by 1.358 ppm, with respect to its position in the spectrum of the free ligand. Similarly as in Cu(Glu)(tn), a small shift (0.042 ppm) of the signal assigned to the C_(5)_ atom excludes from coordination the oxygen atoms from –C_(5)_OO^−^ carboxyl group of Glu. Moreover, the quenching of signals assigned to C_(1)_ and C_(2)_ atoms from Glu in the ^13^NMR spectrum of the complex proves that the oxygen atoms from the –C_(1)_OO^−^ group and the nitrogen atom from the –NH_2_ group from this ligand are involved in the metallation. Therefore, also in Cu(Glu)(Put) the glycine-like coordination of glutamic acid is found, which is strongly confirmed by analysis of changes in the d–d band and EPR spectra (Table 4). This observation confirms that not only the length of diamine carbon chain, but also the length of carbon chain in the amino acid (with respect to the Cu(II)–Asp–diamine system), influence the mode of coordination of the amino acid with Cu(II). The Cu(Glu)(Put)(OH) complex starts forming at pH close to 9 and at pH 10.5 binds almost 60 % of the Cu(II). Similarly as in the spectrum of Cu(Glu)(Put), and also in the NMR spectrum of the hydroxo complex, the signals assigned to C_(1)_ and C_(2)_ of Glu are quenched and the signal assigned to C_(5)_ is shifted only by 0.009 ppm, which points to the glycine-like mode of l-glutamic acid coordination with Cu(II) ions, the same as in the complex with tn. The signal assigned to the C(1) atom from diamine is shifted by 0.214 ppm, which proves that two nitrogen atoms from Put are involved in the metallation, which is also indicated by analysis of the Vis and EPR spectra (Table 4).

## Conclusions

In the metal-free systems, for molecular complexes of glutamic acid with diamines, (Glu)H_*x*_(PA), the interaction centers are the oxygen atoms from carboxyl groups, the nitrogen atom from amine group of amino acid, and the nitrogen atoms from amine groups of diamines. The length of PA chain and the length of the methylene chain in the amino acid have been found to have significant effects on adduct formation. The ability of 1,3-diaminopropane to form stable adducts with l-glutamic acid is greater than that of the one methylene group longer molecule Put, and Glu forms more stable adducts with diamines than aspartic acid [37]. Depending on the pH, the amine groups from a diamine can behave as a negative or positive center of interaction. An inversion effect was found to take place in (Glu)H_2_(tn) adduct, in which the first deprotonated amine group from the diamine acts as a negative center of interaction with a protonated amine group from the amino acid. No inversion effect was found in the system with Put, similar to the system Asp–Put [37], which is related to the differences in the protonation constants of the two polyamines. The centers of noncovalent interactions in the adducts formed in the metal-free systems are also potential sites of metal ion coordination that may affect the mode of interactions taking place between biomolecules. In the Cu(Glu)_2_ complex that forms in the system Cu(II)–Glu, the second molecule of the amino acid coordinates to the anchoring Cu(Glu) with chromophore {N,2O} in a “glycine-like” mode, that is only through two functional groups, while in the earlier studied Cu(Asp)_2_ [68] it occurs through three functional groups. The metal ion interferes in the noncovalent interactions. Introduction of metal ions into the system Glu–diamine changes the character of interactions between bioligands and no molecular complexes are observed to form in the ternary systems. In Cu(II)–Glu–diamine systems the formation of only Cu(Glu)(diamine) and Cu(Glu)(diamine)(OH) complexes was established. It was ascertained that in both complexes the mode of glutamic acid interaction with metal ions is “glycine-like”, which means that only the –NH_2_ amine group and –C_(1)_OO^−^ carboxyl group are involved in the coordination and the second carboxyl group is open to noncovalent interaction with other ligands in biological systems.

## Abbreviations

tn: 1,3-Diaminopropane
Put: 1,4-Diaminobutane, Putrescine
Glu: l-Glutamic acid
PA: polyamine
UV–Vis: UV–Visible spectroscopy
IR: Infrared spectroscopy
TMS: Tetramethylsilane

## References

[1] Attwell D (2000). Brain uptake of glutamate: food for thought. J. Nutr..

[2] Petroff OA (2002). GABA and glutamate in the human brain. Neuroscientist.

[3] Curtis DR, Crawford JM (1969). Central synaptic transmission—microelectrophoretic studies. Ann. Rev. Pharmacol..

[4] Krnjevic K (1970). Glutamate and gamma-aminobutyric acid in brain. Nature.

[5] Logan WJ, Snyder SH (1971). Unique high affinity uptake systems for glycine, glutamic andaspartic acids in central nervous tissue of the rat. Nature.

[6] Vaccarino FM, Schwartz ML, Hartigan D, Leckman JF (1995). Basic fibroblast growthfactor increases the number of excitatory neurons containing glutamate in the cerebralcortex. Cerebr. Cortex.

[7] Meldrum BS (2000). Glutamate as a neurotransmitter in the brain: review of physiology andpathology. J. Nutr..

[8] Bennet MR, Balcar VJ (1999). Forty years of amino acid transmission in the brain. Neurochem. Int..

[9] Monda M, Viggiano A, Sullo A, De Luca V (1998). Aspartic and glutamic acids increase inthe frontal cortex during prostaglandin E1 hyperthermia. Neuroscience.

[10] McEntee WJ, Crook TH (1993). Glutamate: its role in learning, memory, and the agingbrain. Psychopharmacology.

[11] Ozawa S, Kamiya H, Tsuzuki K (1998). Glutamate receptors in the mammalian centralnervous system. Prog. Neurobiol..

[12] Mayer ML, Westbrook GL (1987). The physiology of excitatory amino acids in thevertebrate central nervous system. Prog. Neurobiol..

[13] Meldrum B, Garthwaite J (1990). Excitatory amino acid neurotoxicity and neurodegenerativedisease. Trends Pharmacol. Sci..

[14] Dingledine R, Borges K, Bowie D, Traynelis SF (1999). The glutamate receptor ionchannels. Pharmacol. Rev..

[15] Tapiero H, Mathé G, Couvreur P, Tew KD (2002). Free amino acids in human health andpathologies II. Glutamine and glutamate. Biomed. Pharmacother.

[16] Reeds PJ, Burrin DG, Stoll B, Jahoor F (2000). Intestinal glutamate metabolism. J. Nutr..

[17] Fisher G, Lorenzo N, Ade H, Fujita E, Frey WH, Emory C, Di Fiore MM, D’Aniello A (1998). Free d- and l-amino acids in ventricular cerebrospinal fluid fromAlzheimer and normal subjects. Amino Acids.

[18] Tarbit I, Perry EK, Perry RH, Blessed G, Tomlinson BE (1980). Hippocampal free amino acids in Alzheimer’s disease. J. Neurochem..

[19] Smith CCT, Bowen DM, Francis PT, Snowden JS, Neary D (1985). Putative amino acid transmitters in lumbar cerebrospinal fluid of patients with histologically verified Alzheimer’s dementia. J. Neurol. Neurosurg. Psychiatry.

[20] Hynd MR, Scott HL, Dodd PR (2004). Glutamate-mediated excitotoxcity and neurodegeneration in Alzheimer’s disease. Neurochem. Int..

[21] Rothstein JD (1996). Excitotoxicity hypothesis. Neurology.

[22] Platt SR (2007). The role of glutamate in central nervous system health and disease—a review. Vet. J..

[23] Kusano T, Yamaguchi K, Berberich T, Takahashi Y (2007). Advances in polyamine research in 2007. J. Plant. Res..

[24] Usherwood PNR (2000). Natural and synthetic polyamines: modulators of signaling proteins. Il Farmaco.

[25] Thomas T, Thomas TJ (2001). Polyamines in cell growth and cell death: molecular mechanisms and therapeutic applications. Cell. Mol. Life Sci..

[26] Ouameur AA, Bourassa P, Tajmir-Riahi HA (2010). Probing tRNA interaction with biogenic polyamines. RNA.

[27] D’Agostino L, Di Luccia A (2002). Polyamines interact with DNA as molecular aggregates. Eur. J. Biochem..

[28] Pegg AE (2009). Mammalian polyamine metabolism and function. IUBMB Life.

[29] Jänne J, Alhonen L, Pietilä M, Keinänen TA (2004). Genetic approaches to the cellular functions of polyamines in mammals. Eur. J. Biochem..

[30] Gugliucci A (2004). Polyamines as clinical laboratory tools. Clin. Chim. Acta.

[31] Bachrach U (2004). Polyamines and cancer: minireview article. Amino Acids.

[32] Moinard C, Cynober L, de Bandt JP (2005). Polyamines: metabolism and implications in human diseases. Clin. Nutr..

[33] Paschen W (1992). Polyamine metabolism in different pathological states of the brain. Mol. Chem. Neuropathol..

[34] Morrison LD, Kish SJ (1995). Brain polyamine levels are altered in Alzheimer’s disease. Neurosci. Lett..

[35] Bregier-Jarzebowska R, Gasowska A, Jastrzab R, Lomozik L (2009). Noncovalent interactions and coordination reactions in the systems consisting of copper(II) ions, aspartic acid and diamines. J. Inorg. Biochem..

[36] Bregier-Jarzebowska R, Lomozik L (2010). Noncovalent interactions and copper(II) coordination in systems containing l-aspartic acid and triamines. Polyhedron.

[37] Bregier-Jarzebowska R (2013). Complexes of copper(II) ions with l-aspartic acids in the systems with tetramines and non-covalent interactions between bioligands. J. Coord. Chem..

[38] Tapiero H, Townsend DM, Tew KD (2003). Trace elements in human physiology and pathology. Copper. Biomed. Pharmacother..

[39] Trevors JT, Cotter CM (1990). Copper toxicity and uptake in microorganisms. J. Ind. Microbiol..

[40] Lomozik L (1984). Complex compounds of Cu(II) and Zn(II) with *N*,*N*-dimethylglycine and *N*,*N*-diethylglycine in water and in water–methanol system. Monatsh. Chem..

[41] Irving HM, Miles MG, Pettit LD (1967). A study of some problems in determining the stoichiometric proton dissociation constants of complexes by potentiometric titrations using a glass electrode. Anal. Chim. Acta.

[42] Gans P, Sabatini A, Vacca A (1985). SUPERQUAD: an improved general program for computation of formation constants from potentiometric data. J. Chem. Soc. Dalton Trans..

[43] Gans P, Sabatini A, Vacca A (1996). Investigation of equilibria in solution. Determination of equilibrium constants with the HYPERQUAD suite of programs. Talanta.

[44] Ingri N, Kakolowicz W, Sillén LG, Warnqvist B (1967). High-speed computers as a supplement to graphical methods—V Haltafall, a general program for calculating the composition of equilibrium mixtures. Talanta.

[45] Glasoe PK, Long FA (1960). Use of glass electrodes to measure acidities in deuterium oxide. J. Phys. Chem..

[46] Miranda C, Escarti F, Lamarque L, Yunta MJR, Navarro P, Garcia-España E, Jimeno ML (2004). New 1H-pyrazole-containing polyamine receptors able to complex l-glutamate in water at physiological pH values. J. Am. Chem. Soc..

[47] De Castro M, Lima JLFC, Reis S (1995). Potentiometric determination of formation constants of copper(II)/bile acid/peptide in aqueous solution. J. Pharm. Biomed. Anal..

[48] Martell AE, Smith RM (1982). Critical Stability Constants.

[49] Saroff HA (1998). From glycine to glutamic acid: analysis of the proton-binding isotherm of glutamic acid. Anal. Biochem..

[50] Sylva RN, Davidson MR (1979). The hydrolysis of metal ions Part 1 Copper(II). J. Chem. Soc. Dalton Trans..

[51] Lomozik L, Jaskolski M, Wojciechowska A (1991). A multistage verification procedure for the selection of models in the studies of complex formation equilibria. Pol. J. Chem..

[52] Lomozik L, Jaskolski M, Gasowska A (1995). Comparative analysis of the performance of the computer programs SCOGS, MINIQUAD, and SUPERQUAD in studies of complex- formation equilibria. J. Chem. Educ..

[53] Ramaswamy S, Murthy MRN (1992). Crystal and molecular structures of propanediamine complexed with l-and dl-glutamic Acid: effect of chirality on propanediamine. Acta Cryst..

[54] Wojciechowska A, Bolewski L, Lomozik L (1991). A study of polyamine complex formation with H^+^, Cu(II), Zn(II), Pb(II), and Mg(II) in aqueous solution. Monatsh. Chem..

[55] Lomozik L, Gasowska A (1993). Equilibrium and spectral studies of putrescine complexes with copper(II). Monatsh. Chem..

[56] Lomozik L, Gasowska A, Bolewski L (1997). Noncovalent interactions in polyamine/nucleoside (or diaminocarboxylate) systems studied by potentiometric and NMR techniques. J. Chem. Soc. Perkin Trans..

[57] Lomozik L, Gasowska A (1996). Investigations of binding sites and stability of complexes formed in ternary Cu(II)/adenosine or cytidine/putrescine systems. J. Inorg. Biochem..

[58] Lomozik L, Gasowska A, Krzysko G (2006). Interactions of 1,12-diamino-4,9- dioxadodecane (OSpm) and Cu(II) ions with pyrimidine and purine nucleotides: adenosine-5′-monophpsphate (AMP) and cytidine-5′-monophosphate (CMP). J. Inorg. Biochem..

[59] Lomozik L, Gasowska A (1998). Complexes of copper(II) with spermine and non-covalent interactions in the systems including nucleosides or nucleotides. J. Inorg. Biochem..

[60] Frisch, M.J., Trucks, G.W., Schlegel, H.B., Scuseria, G.E., Robb, M.A., Cheeseman, J.R., Montgomery Jr., J.A., Vreven, T., Kudin, K.N., Burant, J.C., Millam, J.M., Iyengar, S.S., Tomasi, J., Barone, V., Mennucci, B., Cossi, M., Scalmani, G., Rega, N., Petersson, G.A., Nakatsuji, H., Hada, M., Ehara, M., Toyota, K., Fukuda, R., Hasegawa, J., Ishida, M., Nakajima, T., Honda, Y., Kitao, O., Nakai, H., Klene, M., Li, X., Knox, J.E., Hratchian, H.P., Cross, J.B., Bakken, V., Adamo, C., Jaramillo, J., Gomperts, R., Stratmann, R.E., Yazyev, O., Austin, A.J., Cammi, R., Pomelli, C., Ochterski, J.W., Ayala, P.Y., Morokuma, K., Voth, G.A., Salvador, P., Dannenberg, J.J., Zakrzewski, V.G., Dapprich, S., Daniels, A.D., Strain, M.C., Farkas, O., Malick, D.K., Rabuck, A.D., Raghavachari, K., Foresman, J.B., Ortiz, J.V., Cui, Q., Baboul, A.G., Clifford, S., Cioslowski, J., Stefanov, S.S., Liu, G., Liashenko, A., Piskorz, P., Komaromi, I., Martin, R.L., Fox, D.J., Keith, T., Al-Laham, M.A., Peng, C.Y., Nanayakkara, A., Challacombe, M., Gill, P.M.W., Johnson, B., Chen, W., Wong, M.W., Gonzalez, C., Pople, J.A.: Gaussian 03, revision C.02. Gaussian, Inc., Wallingford CT (2004)

[61] Nagypal I, Gergely A, Farkas E (1974). Thermodynamic study of the parent and mixed complexes of aspartic acid, glutamic acid and glycine with copper(II). J. Inorg. Nucl. Chem..

[62] Sajadi SAA (2010). Metal ion-binding properties of l-glutamic acid and l-aspartic acid, a comparative investigation. Nat. Sci..

[63] Bastug AS, Goz SE, Talman Y, Gokturk S, Asil E, Caliskan E (2011). Formation constants and coordination thermodynamics for binary complexes of Cu(II) and some α- amino acids in aqueous solution. J. Coord. Chem..

[64] Gasowska A (2003). Interaction centres of purine nucleotides: adenosine-5′-diphosphate and adenosine-5′-triphosphate in their reactions with tetramines and Cu(II) ions. J. Inorg. Biochem..

[65] Barbucci R, Campbell MJM (1976). An investigation of the structures of copper(II) polyamine complexes in aqueous solution by a combined evaluation of the EPR and theromdynamic parameters. Inorg. Chim. Acta.

[66] Gampp H, Sigel H, Zuberbuechler AD (1982). Apical interactions in copper(II) complexes. Stability and structure of the binary and ternary copper(II) complexes formed with l-alaninamide and diethylenetriamine in aqueous solution. Inorg. Chem..

[67] Gasowska A, Lomozik L, Jastrzab R (2000). Mixex-ligand complexes of copper(II) ions with AMP and CMP in the systems with polyamines and non-covalent interaction between bioligands. J. Inorg. Biochem..

[68] Bregier-Jarzebowska R, Gasowska A, Lomozik L (2008). Complexes of Cu(II) ions and noncovalent interactions in systems with l-aspartic acid and cytidine-5′- monophosphate. Bioinorg. Chem. Appl..

[69] Li NC, Doody E (1952). Metal–aminoacid complexes. II. Polarographic and potentiometric studies on complex formation between copper(II) and aminoacid ions. J. Am. Chem. Soc..

[70] Lomozik L, Bolewski L, Dworczak R (1997). Complex formation in copper(II) ternary systems involving polyamines and diaminocarboxylates studied by potentiometric and spectroscopic techniques. J. Coord. Chem..

